# Rasmussen’s encephalitis: structural, functional, and clinical correlates of contralesional epileptiform activity

**DOI:** 10.1007/s00415-024-12607-7

**Published:** 2024-08-14

**Authors:** Tobias Bauer, Randi D. von Wrede, Suresh Pujar, Attila Rácz, Christian Hoppe, Tobias Baumgartner, Sophia Varadkar, Nina R. Held, Johannes T. Reiter, Selma Enders, Bastian David, Conrad C. Prillwitz, Mar Brugues, Vera C. W. Keil, Monika Jeub, Valeri Borger, Josemir W. Sander, Wolfram S. Kunz, Alexander Radbruch, Bernd Weber, Christoph Helmstaedter, Hartmut Vatter, Torsten Baldeweg, Albert J. Becker, J. Helen Cross, Rainer Surges, Theodor Rüber

**Affiliations:** 1 Department of Neuroradiology, University Hospital Bonn, Bonn, Germany; 2 Department of Epileptology, University Hospital Bonn, Bonn, Germany; 3 Department of Neurology, Great Ormond Street Hospital for Children, London, UK; 4 Developmental Neurosciences Research and Teaching Department, UCL NIHR BRC Great Ormond Street Institute of Child Health, London, UK; 5 Section for Translational Epilepsy Research, Department of Neuropathology, University Hospital Bonn, Bonn, Germany; 6 Department of Radiology and Nuclear Medicine, Amsterdam University Medical Center, Amsterdam, The Netherlands; 7 Amsterdam Neuroscience, Brain Imaging Amsterdam, Amsterdam, The Netherlands; 8 Cancer Center Amsterdam, Imaging and Biomarkers, Amsterdam, The Netherlands; 9 Department of Neurosurgery, University Hospital Bonn, Bonn, Germany; 10 Department of Clinical & Experimental Epilepsy, UCL Queen Square Institute of Neurology, London, UK; 11 Chalfont Centre for Epilepsy, Chalfont St Peter, UK; 12 Department of Neurology, West China Hospital, Sichuan University, Chengdu, China; 13 Institute of Experimental Epileptology and Cognition Research, University Hospital Bonn, Bonn, Germany; 14 Young Epilepsy Lingfield, Lingfield, UK

**Keywords:** Epilepsy, EEG, Neuroimmunology, Neuropsychology, MRI

## Abstract

Progressive inflammation of one hemisphere characterises Rasmussen’s encephalitis (RE), but contralesional epileptiform activity has been repeatedly reported. We aimed to quantify contralesional epileptiform activity in RE and uncover its functional and structural underpinnings. We retrospectively ascertained people with RE treated between 2000 and 2018 at a tertiary centre (Centre 1) and reviewed all available EEG datasets. The temporal occurrence of preoperative contralesional epileptiform activity (interictal/ictal) was evaluated using mixed-effects logistic regression. Cases with/without contralesional epileptiform activity were compared for cognition, inflammation (ipsilesional brain biopsies), and MRI (cortical and fixel-based morphometry). EEG findings were validated in a second cohort treated at another tertiary centre (Centre 2) between 1995 and 2020. We included 127 people with RE and 687 EEG samples. Preoperatively, contralesional epileptiform activity was seen in 30/68 (44%, Centre 1) and 8/59 (14%, Centre 2). In both cohorts, this activity was associated with younger onset age (OR = 0.9; 95% CI 0.83–0.97; *P* = 0.006). At centre 1, contralesional epileptiform activity was associated with contralesional MRI alterations, lower intelligence (*OR* = 5.19; 95% CI 1.28–21.08; *P* = 0.021), and impaired verbal memory (*OR* = 10.29; 95% CI 1.97–53.85;* P* = 0.006). After hemispherotomy, 11/17 (65%, Centre 1) and 28/37 (76%, Centre 2) were seizure-free. Contralesional epileptiform activity was persistent postoperatively in 6/12 (50%, Centre 1) and 2/34 (6%, Centre 2). Preoperative contralesional epileptiform activity reduced the chance of postoperative seizure freedom in both cohorts (*OR* = 0.69; 95% CI 0.50–0.95; *P* = 0.029). Our findings question the concept of strict unilaterality of RE and provide the evidence of contralesional epileptiform activity as a possible EEG predictor for persisting postoperative seizures.

## Introduction

Rasmussen’s encephalitis (RE) is a rare immune-mediated brain disorder characterised by pharmacoresistant focal epilepsy and progressive unihemispheric brain atrophy with neurological deficits and cognitive decline [1, 2]. Since its first description, the unilateral occurrence of RE constitutes its most prominent characteristic [3]. Hemispherectomy, the resection of the affected hemisphere or hemispherotomy, the disconnection of the hemisphere, are currently the only effective treatments. Its success rates support the theory of a strictly unilateral disease [4]. The few existing studies involving EEG recordings in people with RE have repeatedly shown interictal epileptiform activity over the contralesional hemisphere, mainly in an advanced disease stage, and have been related to cognitive decline [5–8]. One study reported interictal epileptiform activity over the contralesional hemisphere in 2/8 (25%) individuals 3–6 months after seizure onset but in 5/8 (62%) individuals 3–5 years after seizure onset. Its occurrence was predictive of a decline in full-scale IQ [6]. Similarly, another study observed the development of contralateral epileptiform activity in 8/12 (66%) individuals after 2 months to 4 years [7]. A third study found interictal epileptiform activity over the contralesional hemisphere in 15/49 (30%) individuals with RE and highlighted the discrepancy between unilateral neurologic signs, MRI, pathologic findings, and bilateral EEG abnormalities [5]. The argument for RE as solely a unihemispheric disease lacks sufficient backing given the paucity of adequately powered EEG studies. Alternative suggestions point towards a potentially more diffuse process, not fully captured by MRI, but this needs confirmation [5, 6].

We hypothesised that contralesional epileptiform activity has structural underpinnings and is functionally and clinically relevant. To examine this hypothesis, we assessed data from two major tertiary epilepsy centres in Europe. We aimed (i) to quantify EEG characteristics in people with RE based on a multimodal dataset, (ii) to link epileptiform EEG activity to MRI, histopathology, neuropsychology, and pre-and postoperative clinical indices, and (iii) to validate the EEG findings on an external dataset.

## Methods

### Study group

**Fig. 1:**
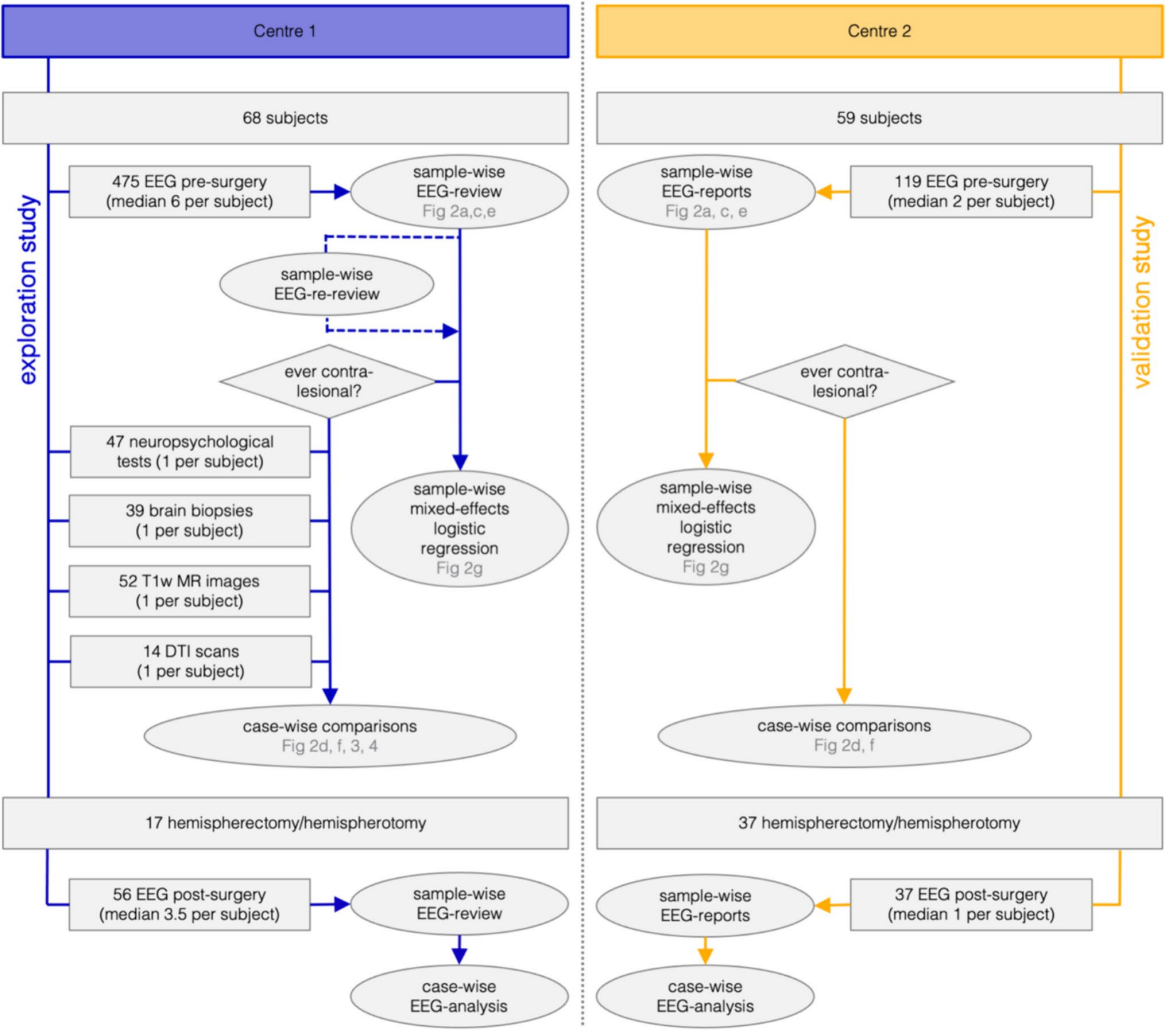
Schematic overview of data and analyses of the study. Data incorporated in the exploratory study at Centre 1 are shown on the left (dark blue arrows and contours), and data used for the validation study at Centre 2 are shown on the right (yellow arrows and contours)

We conducted a retrospective case-note review of people with RE treated between 2000 and 2018 in the Department of Epileptology at the University Hospital Bonn (Centre 1) and treated between 1995 and 2020 at the Great Ormond Street Hospital for Children in London (Centre 2). RE was diagnosed according to the accepted diagnostic criteria [2, 9]. The Bonn cohort was used for exploratory analyses, and the London cohort for validation (Fig. 1). The study was approved by the Institutional Review Board of the University Hospital Bonn and the GOSH clinical audit department as a service evaluation, according to the guidelines set by NHS Research Ethics Committee Review.

### EEG assessment

Awake or asleep scalp EEG was recorded as part of the clinical routine, according to the international 10–20 system. EEG samples at Center 1 were acquired as routine EEG, mobile long-term EEG over 24 h, or video EEG over several days. EEG samples at Centre 2 were acquired as routine EEG or video EEG over several days. EEGs at Centre 1 were recorded using the Stellate Harmonie recording system (Stellate, Montreal, Canada; amplifiers constructed by Schwarzer GmbH, Munich, Germany) initially and from 2013 onwards, the Micromed System S.p.A. (Mogliano, Italy). EEGs were recorded at Centre 2 with a Grass-Telefactor (Astromed, West Warwick, RI, USA) system until 2010, and then with Xltek Trex (Natus Medical Incorporated, Pleasanton, CA, USA). EEG datasets at Centre 1 were reviewed in detail by an experienced senior consultant using a standardised protocol according to clinical standards [10]. To assess inter-rater reliability, a randomly chosen subset of 273 datasets from Centre 1 were re-reviewed by a second experienced senior consultant and discussed in case of disagreement. At Centre 2, EEG findings were extracted from the clinical reports.

Based on either the standardised review (Centre 1) or the clinical reports (Centre 2), data were stratified into three operational categories: Abnormal interictal slowing (ipsilesional, contralesional, or bilateral synchronous), interictal epileptiform discharges (ipsilesional, contralesional, or bilateral synchronous), and ictal patterns (ipsilesional onset, contralesional onset, or bilateral synchronous). We used the more general term *contralesional epileptiform activity* (CEA) if either contralesional interictal epileptiform discharges or contralesional onset of ictal patterns were present. The term *bilateral synchronous* refers to synchronous epileptiform patterns across both hemispheres. In cases where asynchronous epileptiform foci were detected in both hemispheres, they were counted as ipsilesional *and* contralesional.

### Neuropsychological assessment

The test battery for adults used at Centre 1 aligns with European standards [11]. The test battery for children at Centre 1 was designed as parallel [12]. The test ratings result from the integration of standardised scores of single tests that addressed the respective function and were categorised into five operational categories (Strongly impaired—At least two of the test scores for the respective function were at least two standard deviations below the average performance of an age-matched normative sample; Impaired—At least two respective test scores were one standard deviation below average; Borderline—One test score was smaller than one standard deviation below average or at least two test scores roughly equalled average; Average—A maximum of one test score roughly equalled average, while all other scores were average; Above average—At least two test scores were larger than one standard deviation above average).

### MRI acquisition and analysis

At Centre 1, brain MRI acquired using standardised epilepsy protocols [13] at 3 Tesla including non-enhanced T1-weighted sequences with either 1 mm or 0.8 mm isotropic resolution from the latest available MRI scan were selected. To account for the respective contralesional and ipsilesional hemispheres, images of those individuals with a right hemispheric disease focus were flipped along the x-axis. Surface reconstruction and volumetric segmentation were performed using *FreeSurfer* (v6.0, https://surfer.nmr.mgh.harvard.edu/) [14]. Surfaces were registered to a symmetric template for surface-based cross-sectional statistical comparison of cortical thickness and surface area (*fsaverage_sym*). A vertex-wise general linear model using *age at MRI, intracranial volume, ses, MRI resolution,* and *side of disease focus* as covariates was applied for group-level statistical inference. For family-wise error (FWE) correction, cluster mass inference via random-field theory was applied to all results [15]. Additionally, we included diffusion-tensor-imaging (DTI) using either 60 or 32 directions. Preprocessing and fixel-based analysis using *MRtrix3* (v3.0.4, https://www.mrtrix.org/) [16, 17] were performed as described previously [18]. For fixel-based cross-sectional statistical comparison of fibre density and cross-section, all fixel-images were registered to a symmetric group-specific template. A fixel-wise general linear model using *age at MRI, sex,* and *DTI sequence* as covariates was applied for group-level statistical inference. Due to the small sample size, no FWE correction was applied.

### Neuropathological markers of neuroinflammation

Neuropathological examinations of brain biopsies from the ipsilesional hemisphere performed as part of the clinical assessment at Centre 1 were conducted as described elsewhere [19, 20], including standard histological staining as well as immunohistochemistry panels for T- and B-lymphocytes, plasma cells, and activated microglial cells. See related previous publications [19–22] for methodological details.

### Statistical analysis

In sample-wise mixed-effects logistic regression analyses (findings from every single EEG examination separately), the presence of CEA was set as the dependent variable, *age at disease onset*, *disease duration, lesional hemispheric side,* and *sex* were included as covariates. In outcome-related case-wise analyses (findings based on all available EEG examinations per case, see Fig. 1), seizure freedom at last follow-up after hemispherotomy was set as the dependent variable, the *presence of presurgical CEA*, *age at disease onset*, *disease duration at hemispherotomy, lesional hemispheric side,* and *sex* were set as covariates*.* When both datasets were analysed separately in sample-wise analyses, the *subject* was added as a random effect. When the combined datasets were analysed, *subject* was nested within *site* as a random effect. Categorical data presented in contingency tables were analysed using Fisher’s exact test. Strengths of associations are presented as odds ratios with 95% confidence intervals. Distributions of categorical data were compared using Chi-square tests with Yate’s correction for continuity. Inter-rater reliability was assessed using Cohen’s kappa. The means of two groups were compared using Wilcoxon rank-sum tests. All statistical analyses were performed using freely available *SciPy*, *statsmodels*, and *Pymer4* modules for Python. An effect is regarded statistically significant if *P* < 0.05.

## Results

### Clinical characteristics of exploration cohort at Centre 1

At Centre 1, 68 individuals with RE (39 female, 36 left-hemispheric focus, median onset seven years, range 1–51 years) met the diagnostic criteria for RE and were included in this study (Table 1). Brain biopsies that confirmed RE diagnosis were performed in 39/68 (57%) cases. All individuals, except for two, were treated with anti-seizure medication (median number of anti-seizure medication taken as monotherapy or in combinations in the past history 6, range 0–15). Fifty-nine subjects (87%) received at least one type of immunotherapy. Seventeen individuals (25%, median age at onset 5 years, range 2–11 years) underwent hemispherotomy, and postsurgical EEG data were available in 12 cases. No cases of incomplete disconnection were identified in the neuroradiological reviews of postoperative MRI.

**Table 1:** Overview of data analysed in this study

	Centre 1	Centre 2	*P*
Subjects; *n*	68	59	n/a
Sex female; *n* (%)	39/68 (57%)	35/59 (59%)	0.96
Left focus; *n* (%)	36/68 (53%)	33/59 (56%)	0.87
Age at onset (years); median (range)	7 (1–51)	6 (2–14)	0.072
Childhood onset, < 10 years; *n* (%)	42 (62%)	51 (86%)	0.002
Adolescence onset, 10–19 years; *n* (%)	15 (22%)	8 (14%)	0.21
Adult onset, > 19 years; *n* (%)	11 (16%)	0	n/a
EEG samples total; *N*	531	156	n/a
EEG hours total (h)	2882.2	3503.2	n/a
EEG hours per subject (h); median (range)	54.4 (1.5–254)	46.5 (0.5–288.5)	0.43
Presurgical EEG total; *N*	475	119	n/a
Presurgical EEG per subject; median (range)	6 (1–36)	2 (1–5)	< 0.001
Disease duration at presurgical EEG (years); median (range)	6 (0–54)	3 (0–12)	< 0.001
Number of anti-seizure medication per subject^a^	6 (0–15)	6 (2–14)	0.86
Immunotherapy; *n* (%)	59 (87)	53 (89)	0.80
Hemispherotomy; *n* (%)	17/68 (25%)	37/59 (63%)	< 0.001
Childhood onset and hemispherotomy; *n* (%)	16/42 (38%)	33/51 (65%)	0.019
Adolescence onset and hemispherotomy; *n* (%)	1/15 (7%)	4/8 (50%)	0.06
Adult onset and hemispherotomy; *n* (%)	0	0	n/a
Hemispherotomy with postsurgical EEG; *n* (%)	12/68 (18%)	34/59 (58%)	< 0.001
Postsurgical EEG total; *N*	56	37	n/a
Postsurgical EEG per subject; median (range)	3.5 (1–12)	1 (1–3)	< 0.001
Disease duration at postsurgical EEG (years); median (range)	11 (2–42)	6 (1–14)	< 0.001
Age at hemispherotomy (years); median (range)	10 (3–46)	11 (3–18)	0.46
Disease duration at hemispherotomy (years); median (range)	4 (0–40)	4 (0–11)	0.90
Last follow-up after hemispherotomy (years); median (range)	4.5 (1–8)	2 (0–7)	0.076
Biopsies; *n* (%)	39/68 (57%)	n/a	n/a
Disease duration at biopsy (years); median (range)	2 (0–42)	n/a	n/a
MRI scans; *n* (%)	52/68 (76%)	n/a	n/a
Disease duration at MRI (years); median (range)	6 (0–43)	n/a	n/a
Diffusion-tensor-imaging; *n* (%)	14/68 (21%)	n/a	n/a
Disease duration at diffusion-tensor-imaging (years); median (range)	8·5 (0–42)	n/a	n/a
Neuropsychological tests; *n* (%)	47/68 (69%)	n/a	n/a
Disease duration at neuropsychological test (years); median (range)	5 (0–43)	n/a	n/a

*P* values refer to Wilcoxon’s rank-sum tests (ordinal data) or Chi-square tests (categorial data), n/a: not applicable

^a^Taken as monotherapy or in combinations in the past history

### Clinical characteristics of validation cohort at Centre 2

The validation cohort from Centre 2 consists of 59 individuals with RE (35 female, 33 left-hemispheric focus, median onset 6 years, range 2–14 years). Brain biopsies were performed in 12/59 (20%) subjects. All individuals were treated with anti-seizure medication (median number of anti-seizure medication 6, range 2–14). Fifty-three subjects (89%) received at least one type of immunotherapy. Thirty-seven individuals (63%, median age at onset 5 years, range 2–14 years) from London underwent hemispherotomy, with postsurgical EEG available in 34 cases. The use of anti-seizure medication (*U* = 1708.5, *P* = 0.86) and immunotherapy (χ^2^(1) = 0.29, *P* = 0.80) did not differ between both centres. Significantly more individuals were treated surgically at Centre 2 than at Centre 1 (χ^2^(1) = 16.9, *P* < 0.001).

### EEG findings in exploration study

**Fig. 2:**
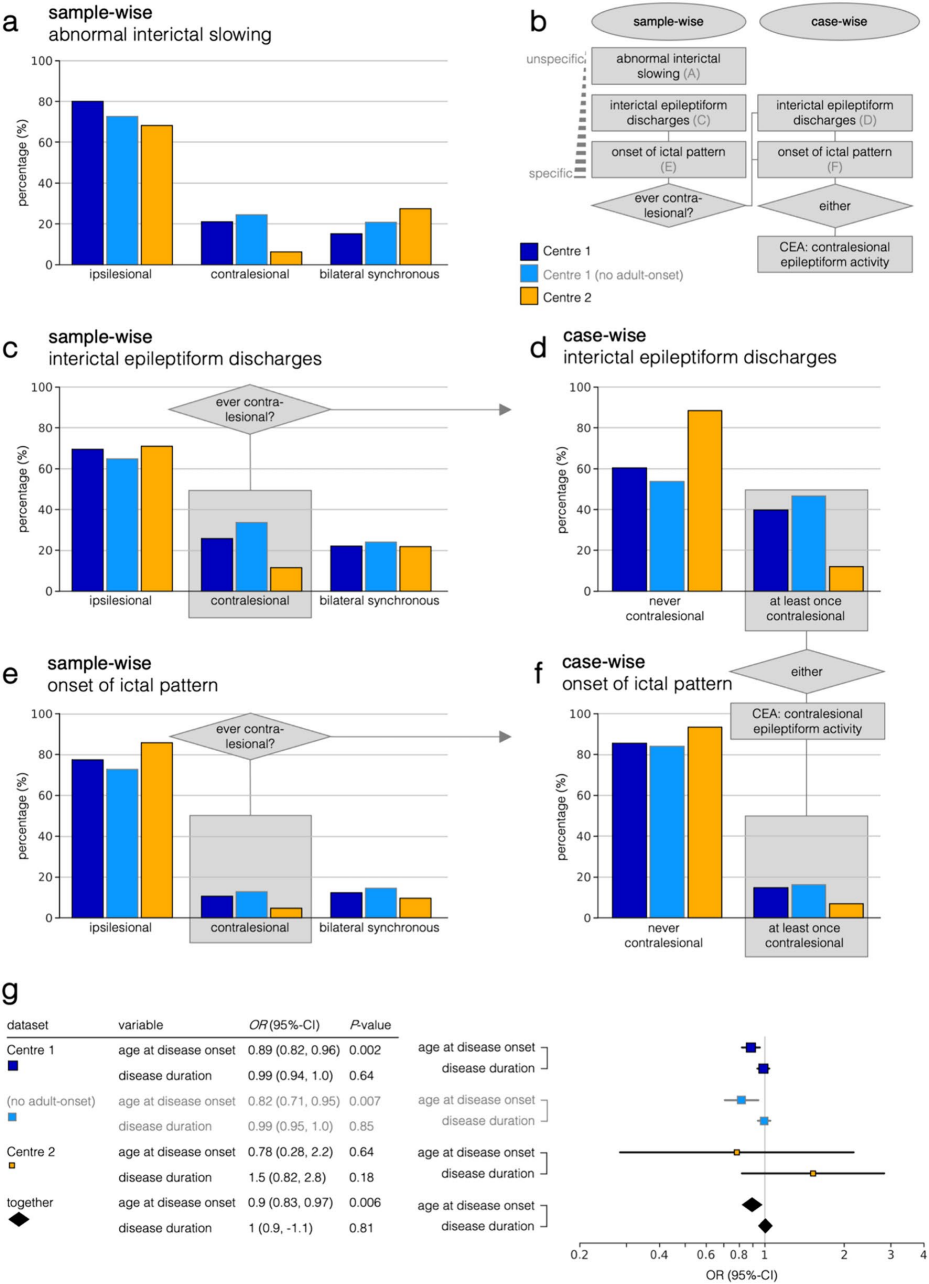
Summary of sample-wise and case-wise EEG findings. **b** Schematic overview over sample-wise and case-wise data representation in this study. In case-wise analyses, only those individuals who showed CEA in at least one sample were included (*bilateral synchronous* refers to synchronous epileptiform patterns across both hemispheres. When asynchronous epileptiform patterns were detected in both hemispheres, they were counted as ipsilesional *and* contralesional; data add up to > 100%, since single EEG samples may contain independent, and therefore not bilateral synchronous, foci in ipsilesional and contralesional hemispheres): **a** interictal slowing, **c** interictal epileptiform discharges, and **e** onset of ictal pattern. Percentages of individuals in whom contralesional patterns were seen in the respective categories: **d** interictal epileptiform discharges, **f** onset of ictal pattern. **g** Sample-wise mixed-effects logistic regression results are shown for Centre 1(top, dark blue marker), Centre 1 without adult-onset cases (second row, light blue marker), Centre 2 (third row, yellow marker), and across both groups (bottom, black marker). EEG was set as the dependent variable. Age at disease onset, disease duration, sex, and lesional side were included as covariates. The subject was added as a random effect on datasets separately

For the exploration study at Centre 1, a total of 2882.2 EEG recording hours in 531 samples (56/531 post-hemispherotomy) were analysed, with a median of 54.4 EEG recording hours per subject. In sample-wise analysis of presurgical EEG, contralesional interictal epileptiform discharges were detected in 58/226 (26%) EEG samples with interictal epileptiform discharges (Fig. 2c, dark blue bars). Contralesional seizure onset was found in 18/172 (10%) EEG samples with seizure patterns (Fig. 2e, dark blue bars). When adult-onset cases were excluded, contralesional interictal epileptiform discharges were present in 57/170 (66%) EEG samples with interictal epileptiform discharges (Fig. 2c, light blue bars). Contralesional seizure onset was observed in 17/132 (13%) EEG samples with seizure patterns (Fig. 2e, light blue bars). In case-wise analyses, CEA (at least one EEG sample with contralesional interictal epileptiform discharges or contralesional ictal onset) was found in 30/68 (44%) individuals (Fig. 2d, f, dark blue bars). When adult-onset cases were excluded, CEA was observed in 28/56 (50%) individuals (Fig. 2d, f, light blue bars). There was no significant difference regarding total EEG recording hours between cases with CEA and cases without CEA (*U* = 216, *P* = 0.66; without adult-onset cases *U* = 165, *P* = 0.14). Inter-rater reliability was *κ* = 0.19 for the detection of abnormal interictal slowing, *κ* = 0.44 for interictal epileptiform discharges, *κ* = 0.53 for ictal patterns, and *κ* = 0.39 (fair agreement) averaged across all categories.

### EEG findings in validation study

For the validation study at Centre 2, a total of 3503.2 EEG recording hours in 156 samples (37/156 post-hemispherotomy) were included, with a median of 46.5 EEG recording hours per subject. In sample-wise analysis of presurgical EEG, contralesional interictal epileptiform discharges were detected in 13/112 (12%) EEG samples with interictal epileptiform discharges (Fig. 2c, yellow bars). Contralesional seizure onset was found in 4/79 (5%) EEG samples with seizure patterns (see Fig. 2e). In case-wise analyses, CEA was found in 8/59 (14%) individuals (Fig. 2d, f, yellow bars). There was no significant difference in total EEG recording hours between cases with CEA and those without CEA (*U* = 254, *P* = 0.27). The frequency distributions between both centres did not differ significantly about interictal epileptiform discharges (χ^2^(2) = 5.82; *P* = 0.055) and ictal onset (χ^2^(2) = 2.99; *P* = 0.22), but concerning abnormal interictal slowing (χ^2^(2) = 19.60; *P* < 0.001). The frequency distributions between the cohort from Centre 1 without adult-onset cases (childhood and adolescence cases only, onset < 19 years) and the cohort from Centre 2 only showed a trend-level difference regarding ictal onset (χ^2^(2) = 5.57, *P* = 0.062), but were significantly different concerning abnormal interictal slowing (χ^2^(2) = 13.90, *P* < 0.001) and interictal epileptiform discharges (χ^2^(2) = 12.65, *P* = 0.002).

### Mixed effects logistic regression of contralesional epileptiform activity

In a sample-wise mixed-effects logistic regression model across both centres, the presence of CEA was significantly associated with a younger age at onset (*OR* = 0.9; 95% CI 0.83–0.97; *P* = 0.006), but not disease duration (*OR* = 1.0; 95% CI 0.95–1.1; *P* = 0.81) (Fig. 2g). When analysing interictal and ictal patterns separately, both interictal epileptiform discharges (*OR* = 0.90; 95% CI 0.84–0.98; *P* = 0.011) and ictal onset (*OR* = 0.83; 95% CI 0.64–1.1; *P* = 0.19) were more likely with younger age at onset; however, the latter effect did not reach statistical significance. From this analysis, however, it cannot be said whether contralesional interictal or ictal patterns ooccur first.

### Post-hemispherotomy outcome

At Centre 1, 11/17 (65%; 7/12 [58%] with preoperative CEA, 4/5 [80%] without preoperative CEA) and at Centre 2, 28/37 (76%; 3/5 [60%] with preoperative CEA, 25/32 [78%] without preoperative CEA) were seizure-free after hemispherotomy. ILAE outcomes (1-2-3-4-5-6) at Centre 1 were 7-0-2-2-1-0/12 in cases with preoperative CEA (7 with ILAE outcome 1; 0 with ILAE outcome 2; 2 with ILAE outcome 3;…), and 4-0-1-0-0-0/5 in cases without preoperative CEA. At Centre 2, ILAE outcomes were 3-0-0-2-0-0/5 in preoperative CEA cases and 23-0-5-3-1-0/32 in cases without preoperative CEA. In a mixed-effects logistic regression model across both centres, becoming seizure-free after hemispherotomy was significantly less likely if CEA was present before surgery (*OR* = 0.69; 95% CI 0.50–0.95; *P* = 0.029). When analysing interictal and ictal patterns separately, postoperative seizure freedom was significantly less likely if preoperative contralesional interictal epileptiform discharges were present (*OR* = 0.70; 95% CI 0.50–0.98; *P* = 0.045), but not associated with preoperative contralesional ictal onset (*OR* = 0.91; 95% CI 0.57–1.45; *P* = 0.70). Contralesional interictal epileptiform discharges persisted in 6/12 (50%, Centre 1) and 2/34 (6%, Centre 2) of all hemispherotomy cases with postoperative EEG available.

### Results of morphometric MRI analysis

**Fig. 3:**
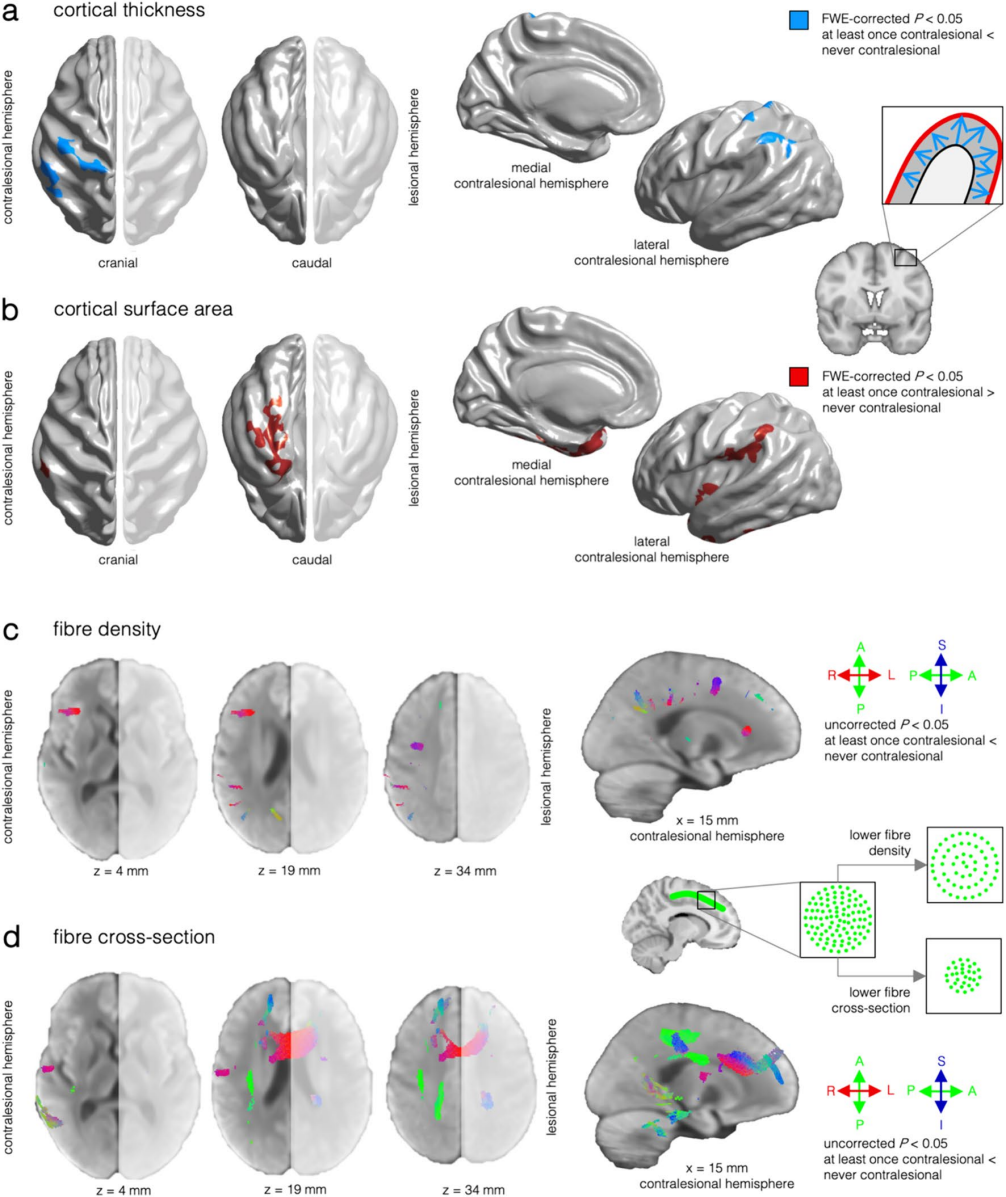
Results of MRI morphometry. **a** Significant clusters (FWE-corrected *P* < 0.05) indicating lower cortical thickness in individuals with CEA in at least one EEG sample as compared to individuals without CEA were observed in the temporoparietal junction and postcentral gyrus (blue). **b** Significant clusters (FWE-corrected *P* < 0.05) indicating higher cortical surface area in individuals with CEA located in the temporoparietal junction, insular cortex, and temporal pole (red). Significant clusters in (**a**, **b**) are visualised on the *fsaverage_sym* template. **c** Visualisation of fibres with lower (uncorrected *P* < 0.05) fibre density in the periinsular and parietal white matter in the contralesional hemisphere of individuals with CEA. **d** Display of fibres with lower (uncorrected *P* < 0.05) fibre cross-section in the anterior body of the corpus callosum, the superior longitudinal fascicle, and the cingulum of the contralesional hemisphere, as well as in the pyramidal tract of the lesional hemisphere in individuals with CEA. Thresholded streamlines in (**c**, **d**) are shown on the study-specific template; grey values represent the magnitude of the fibre orientation distributions. A: anterior, I: inferior, L: left, P: posterior, R: right, S: superior

T1-weighted MRI scans of 52 subjects and DTI scans of 14 subjects from Centre 1 were included. We observed significantly (FWE-corrected *P* < 0.05) lower cortical thickness of the temporoparietal junction and postcentral gyrus (Fig. 3a), as well as higher surface area of the insular cortex, temporoparietal junction and temporal pole of the contralesional hemisphere in individuals who presented at least once with CEA than in individuals who never presented with CEA (Fig. 3b). There was no significant difference between cases with CEA and without CEA included in the surface-based analysis regarding disease duration at MRI (*U* = 423.5, *P* = 0.94), number of anti-seizure medication (*U* = 304, *P* = 0.69), and immunotherapy (χ^2^(1) = 0.99, *P* = 0.32).

Regarding white matter integrity, we observed lower (uncorrected *P* < 0.05) fibre density in the contralesional parietal and periinsular white matter (Fig. 3c). We found lower fibre cross-section in the anterior body of the corpus callosum, the superior longitudinal fascicle, and the cingulum of the contralesional hemisphere, as well as in the pyramidal tract of the lesional hemisphere in individuals with CEA as compared to individuals without CEA (Fig. 3d). There was no significant difference between cases with CEA and without CEA included in the fixel-based analysis regarding disease duration at MRI (*U* = 12.5, *P* = 0.20), the number of anti-seizure medication (*U* = 27, *P* = 0.59), and immunotherapy (χ^2^(1) = 1.75, *P* = 0.19).

### Neuroinflammation

In the semiquantitative approach, inflammation was classified as weak-strong in 4-18 individuals with CEA (4 with weak inflammation; 18 with strong inflammation) and 1-16 individuals without CEA. T-lymphocytes were classified as few-intermediate-strong-perineuronal in 1-5-6-10 individuals with CEA and 0-2-6-9 in individuals without CEA. Microglia were classified as few-intermediate-strong-nodules in 0-4-6-12 individuals with CEA and 0-2-5-10 in individuals without CEA. We did not observe any significant group difference in markers of inflammation (*OR* = 0.28; 95% CI 0.028–2.78; *P* = 0.28), T-lymphocytes (*OR* = 0.25; 95% CI 0.062–2.04; *P* = 0.25), or microglia count (*OR* = 0.60; 95% CI 0.096–3.74; *P* = 0.58) in ipsilesional brain biopsies between individuals who had at least once CEA and individuals who never showed CEA.

### Neuropsychological performance

**Fig. 4:**
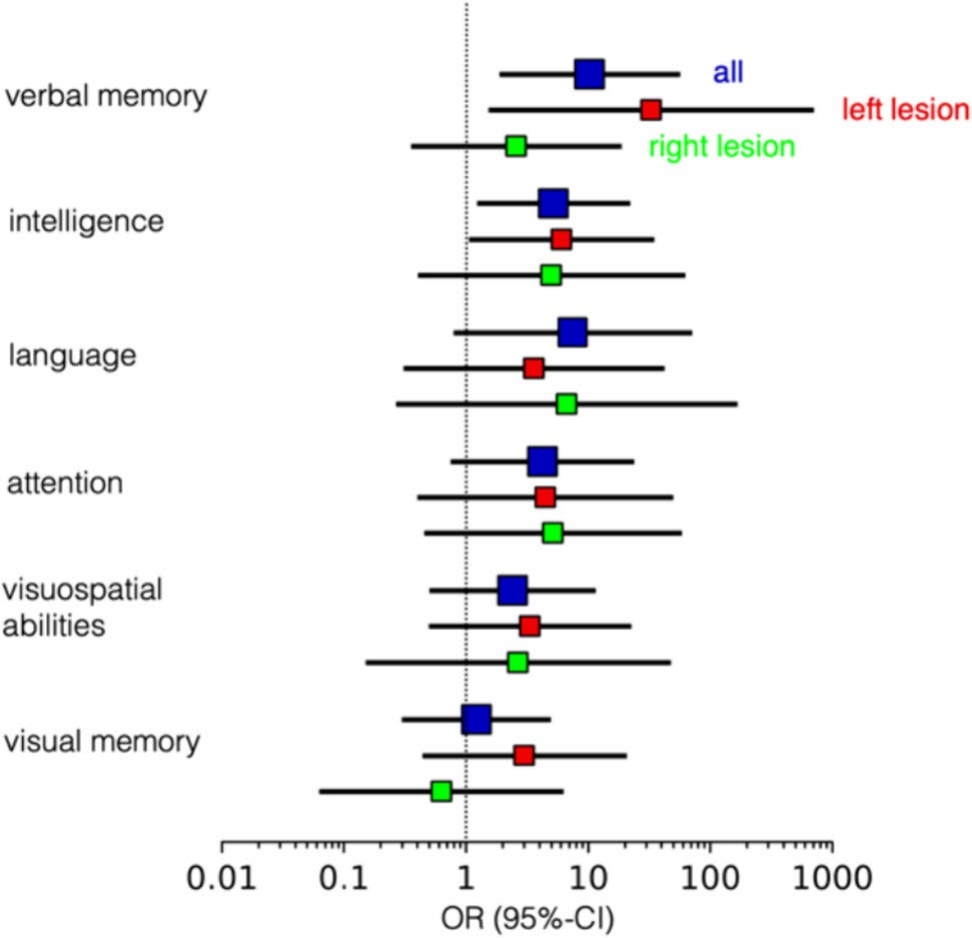
Neuropsychological deficit in relation to the presence of contralesional epileptiform activity. Odds ratios for neuropsychological deficits when CEA is present were evaluated for six cognitive domains. Odds ratios were calculated for all people with Rasmussen’s encephalitis (*n* = 47, dark blue markers), as well as for individuals with left lesional hemispheres (*n* = 25, red markers) and right lesional hemispheres (*n* = 22, green markers) separately

Neuropsychological assessment at Centre 1 was available for 47 people with RE. Individuals with CEA performed significantly worse (strongly impaired, impaired, or borderline) than individuals who never showed CEA concerning intelligence (*OR* = 5.19; 95% CI 1.28–21.08; *P* = 0.021) and verbal memory (*OR* = 10.29; 95% CI 1.97–53.85; *P* = 0.006). While this equally holds for individuals with either left or right lesional hemispheres regarding intelligence, the finding relating to verbal memory is mainly driven by individuals with lesions in the left hemispheres. A general trend is confirmed in all other cognitive domains (attention, visual memory, visuospatial abilities, and language): More individuals with CEA were impaired within the respective category than individuals without CEA (Fig. 4).

## Discussion

Based on a dataset from two epilepsy centres, CEA was observed in almost half of the cases at Centre 1 and in less than a sixth at Centre 2. CEA was associated with younger age at disease onset, persisted after hemispherotomy, and was associated with an increased risk of unfavourable postsurgical seizure outcome. In the exploration study conducted at Centre 1, it was also related to altered morphometry in temporal and insular cortices, with lower intelligence and worse verbal memory.

Frequencies of EEG findings on a sample-wise level did not differ significantly in both centres. On a case-wise level, however, CEA was found in almost half of all individuals at Centre 1, but in less than a sixth at Centre 2. The discrepancy between centres may be explained by the lower number of EEGs per case at Centre 2. Another explanation for the difference is the study design: EEG findings were based on a detailed expert review of the raw data at Centre 1. Consensus was reached in case of disagreement, which can be regarded as an academic gold standard. In contrast, EEG findings were directly extracted from routine clinical reports at Centre 2, corresponding to daily clinical practice. In this reading, this difference suggests that subtle CEA may be overlooked in routine EEG assessments. To address this issue, it may be appropriate to analyse the raw EEG data objectively in future studies using computer-aided approaches.

Our study indicates that postoperative seizures seem to be more likely when CEA is present preoperatively. CEA could be used as a marker for predicting postsurgical seizure freedom. It should not be dismissed that approximately a quarter of individuals with RE are not seizure-free postoperatively [4]. As our data do not show the progression of CEA during the disease course, this may suggest that individuals with RE and CEA are less likely to benefit from surgery from the outset. This finding, however, needs to be validated in prospective cohorts.

On clinical grounds, neuropsychology demonstrates the functional relevance of CEA. It has previously been noted that mental impairment seems to correlate with the bilateral occurrence of EEG epileptic abnormalities in RE, which aligns with our results [8].

Regarding the neuropathological findings, it cannot be said with absolute confidence whether the lack of a group difference in inflammatory markers between individuals with and without CEA represents a lack of association or merely a sampling error. A brain biopsy in one of the contralesional regions indicated by the MRI analyses would be of utmost interest but cannot be ethically justified in typical, unilateral RE cases. In a recent case report of an individual with bilateral RE and bilateral imaging findings [23], biopsies were taken from the presumed inflammatory hotspots in both hemispheres. Twenty-five months after disease onset, inflammation indicative of RE was observed only in the primarily affected hemisphere. However, eighteen months later, histopathological findings consistent with RE were found in biopsies from both hemispheres. This demonstrates that RE-typical inflammation can indeed be present in both hemispheres.

While the conventional MRI reading in typical (non-bilateral) RE yields an “unaffected” contralesional hemisphere, investigations applying quantitative image analyses to a collective of people with RE have shown lower and higher cortical volume of the contralesional hemisphere than controls [24–27]. In our study, analyses of between-group differences between individuals with and without CEA showed lower cortical thickness and higher surface area in distinct regions of the contralesional hemisphere in individuals with CEA. Cortical thickness is commonly interpreted as a proxy for the number of cells within a cortical column and is usually associated with progressive degenerative processes. The surface area, however, reflects cellular-level processes perpendicular to cortical columns. Our finding of a larger surface area could be explained by cortical misfolding as a sequelae of cortical maldevelopment [28, 29]. Of note is that the insula is the preferred site for the primary lesion of the ipsilesional hemisphere, where mild cortical atrophy is often first observed [30, 31]. Our imaging findings in the contralesional hemisphere, thus, affect the homotopic region. The reduced microstructural integrity of the contralesional white matter tracts in individuals with CEA is most likely a morphometric correlate of antecedent epileptic activity. However, as the microstructural alterations affect the entire contralesional white matter and involve intrahemispheric association fibres, this argues against a mere spread of epileptiform activity from the lesional hemisphere.

Interpreting our results is challenged by the limits associated with any retrospective and multicentric study. Diagnostic and therapeutic approaches have changed during the 18 years (Centre 1) or the 25 years (Centre 2) collecting periods, which may bias our results. There are considerable differences between the cohorts from both centres, including the proportion of subjects who underwent hemispherotomy, the age at which they did, and the approach to EEG analysis. Finally, the association between CEA and postoperative seizure outcome was not statistically significant for either centre separately but significant when combined. This could be due to lower power when analysing only one cohort and underlines the value of the joint analysis of multicentric data. Regarding our MRI findings in the insula, it must be considered that EEG allows only poor localisation, especially in the frontal lobe and the insula, so it is technically unfeasible to align the EEG alterations with the imaging findings spatially [32]. Finally, we acknowledge that an incomplete disconnection, indicative of surgical failure, may partially explain the presence of postoperative contralesional epileptiform activity.

At the core of our study lies the compelling issue of the strict unilaterality of RE [1, 33]. While limited by the inherent problems of a retrospective design and the clinical differences between the two centres, we confirm the occurrence of CEA and show the associated MRI, clinical and neuropsychological features. Together with the fact that approximately a quarter of people with RE are not seizure-free post-hemispherotomy [4], our study casts doubt on the paradigm of RE as a strictly unihemispheric disease. While this study cannot determine the nature of contralesional involvement, we conclude from this and previous studies [6, 24, 25, 34] that even though the primary pathological process may be confined to one cerebral hemisphere, RE as a neurological disease is not. The following steps to scrutinise the contralesional hemisphere’s involvement in RE include additional cohorts from other centres, the joint analysis of different diagnostic modalities (e.g., positron-emission tomography) and advanced modelling of MRI data (e.g., disease epicentre mapping). The next logical step is to validate the insights gained from this study using automated EEG analysis. The most promising approach would be a biopsy in one of the contralesional regions. However, this is only ethically justifiable in the case of noticeable and poorly understood structural abnormalities, which, as we show, are only subtle at the individual level.

## Data Availability

The data that support the findings of this study are available on request from the corresponding author. The data are not publicly available due to privacy or ethical restrictions.
